# Supplementary material for: Effects of Cortactin Expression on Prognosis in Patients with Breast Cancer
Source: Diagnostics (Basel). 2023 Sep 7;13(18):2876. doi: 10.3390/diagnostics13182876 (PMC10530131; doi:10.3390/diagnostics13182876)
Supplement: Supplementary file 1 [file diagnostics-13-02876-s001.zip › diagnostics-2563277-supplementary.pdf]

Supplementary Table S1. Correlations between cortactin expression and clinicopathological factors in the triple-negative subtype of breast cancer (n = 108).

Parameter		CTTN expression		p Value
		High group (n=71) No. (%)	Low group (n=37) No. (%)	
Age				0.964 †
		51 ± 10.5	50.6 ± 11	
Tumor size				0.775 †
		3.0 ± 2.2	3.3 ± 2.5	
Histologic grade				0.565
	Grade 1	6 (8.45%)	3 (8.11%)	
	Grade 2	13 (18.31%)	10 (27.03%)	
	Grade 3	52 (73.24%)	24 (64.86%)	
pT stage				0.548
	T1	18 (25.35%)	12 (32.43%)	
	T2	46 (64.79%)	19 (51.35%)	
	T3	4 (5.63%)	4 (10.81%)	
	T4	3 (4.23%)	2 (5.41%)	
pN stage				0.928
	N0	46 (64.79%)	23 (62.16%)	
	N1	15 (21.13%)	7 (18.92%)	
	N2	4 (5.63%)	3 (8.11%)	
	N3	6 (8.45%)	4 (10.81%)	
AJCC * stage (8th)				0.637
	I	15 (21.13%)	9 (24.32%)	
	II	43 (60.56%)	19 (51.35%)	
	III	13 (18.31%)	9 (24.32%)	

*AJCC, American Joint Committee on Cancer; SD, standard deviation; † Mann–Whitney U test.

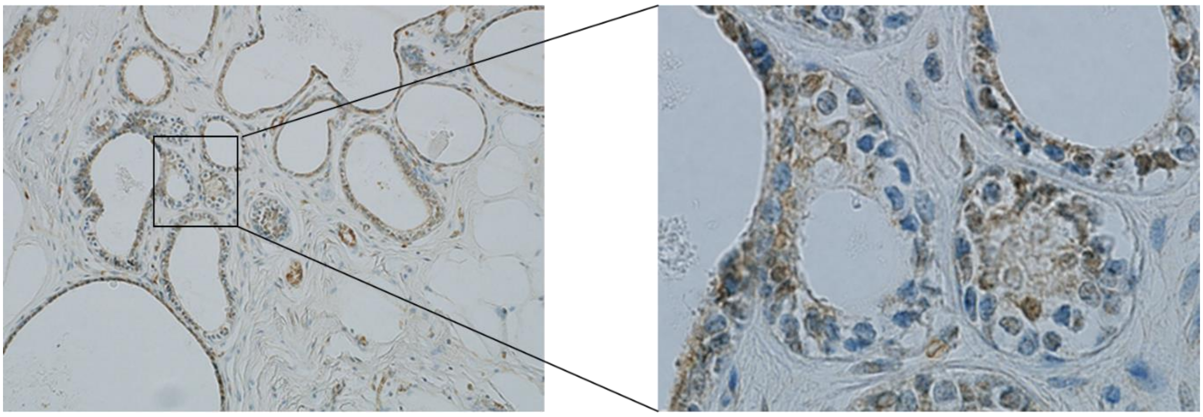

Supplementary Figure S1 Representative photomicrographs of normal tissue, original magnification 200×; original magnification 1000×.
